# Prevalence of non-communicable chronic diseases: arterial hypertension, diabetes mellitus, and associated risk factors in long-lived elderly people

**DOI:** 10.1590/0034-7167-2022-0592

**Published:** 2023-10-09

**Authors:** Andreia Matos da Silva, Ariane Silva do Carmo, Vicente Paulo Alves, Luiz Sérgio Fernandes de Carvalho

**Affiliations:** I Universidade Católica de Brasília. Brasília, Distrito Federal, Brazil

**Keywords:** Disease, Aged, Octogenarians, Risk Factors, Prevalence., Enfermedad, Anciano, Anciano de 80 o Más Años, Factores de Riesgo, Prevalencia., Doença, Idoso, Idoso de 80 Anos ou Mais, Fatores de Risco, Prevalência.

## Abstract

**Objective:**

To identify the prevalence of non-communicable chronic diseases: arterial hypertension, diabetes mellitus, and associated risk factors in long-lived elderly people from three Brazilian regions.

**Methods:**

This is a multicenter, cross-sectional, and comparative study conducted with elderly people aged 80 years or older.

**Results:**

Higher prevalence of arterial hypertension were observed among those who use polypharmacy (75.7%), among elderly people aged between 80 and 84 years (33.9%), as well as in elderly people who are overweight (78.2%). The prevalence of diabetes was 24% (RP: 0.76; 95% CI: 0.59-0.98) lower among women compared to men and 2.15 times higher among those who use five or more medications (RP: 2.15; 95% CI: 1.63-2.85).

**Conclusions:**

In our sample, polypharmacy, body weight, and gender determine the prevalence of non-communicable chronic diseases: arterial hypertension and diabetes mellitus in long-lived elderly people.

## RESUMO

**Objetivo:**

identificar a prevalência das doenças crônicas não transmissíveis: hipertensão arterial, diabetes mellitus e os fatores de risco associados em pessoas idosas longevas de três regiões brasileiras.

**Métodos:**

trata-se de um estudo multicêntrico, transversal e comparativo, realizado com pessoas idosas com idade igual ou superior a 80 anos. **Resultados: f**oram observadas maiores prevalências de hipertensão arterial entre aqueles que fazem uso de polifarmácia (75,7%), entre as pessoas idosas com idade entre 80 e 84 anos (33,9%), bem como em pessoas idosas que apresentam sobrepeso (78,2%). A prevalência de diabetes foi 24% (RP: 0,76; IC 95%: 0,59-0,98) menor entre as mulheres quando comparadas aos homens e 2,15 vezes maior entre aqueles que utilizam cinco ou mais medicamentos (RP: 2,15; IC 95%: 1,63-2,85).

**Conclusões:**

Em nossa amostra, a polifarmácia, o peso corporal e o sexo determinam a prevalência das doenças crônicas não transmissíveis: hipertensão arterial e diabetes mellitus em pessoas idosas longevas.

## RESUMEN

**Objetivo:**

Identificar la prevalencia de enfermedades crónicas no transmisibles: hipertensión arterial, diabetes mellitus y los factores de riesgo asociados en ancianos longevos de tres regiones brasileñas.

**Métodos:**

Se trata de un estudio multicéntrico, transversal y comparativo, realizado con ancianos con edad igual o superior a 80 años.

**Resultados:**

Se observaron mayores prevalencias de hipertensión arterial entre aquellos que hacen uso de polifarmacia (75,7%), entre los ancianos con edad entre 80 y 84 años (33,9%), así como en ancianos que presentan sobrepeso (78,2%). La prevalencia de diabetes fue 24% (RP: 0,76; IC 95%: 0,59-0,98) menor entre las mujeres en comparación con los hombres y 2,15 veces mayor entre aquellos que utilizan cinco o más medicamentos (RP: 2,15; IC 95%: 1,63-2,85).

**Conclusión:**

En nuestra muestra, la polifarmacia, el peso corporal y el sexo determinan la prevalencia de las enfermedades crónicas no transmisibles: hipertensión arterial y diabetes mellitus en ancianos longevos.

## INTRODUCTION

Brazil is aging, and this is justified by the decline in fertility rates associated with the increase in life expectancy^(1)^. In addition, the population of very old elderly individuals (age 80 years and over) is expected to reach significant numbers of 434 million by the year 2050, tripling the quantity compared to 2015^(1-2)^. In this context, non-communicable chronic diseases (NCDs) related to aging represent a relevant global public health problem, causing family, social and economic impacts^(3-4)^.

In the age group of 75 years, for example, in the year 2020 in Brazil, 67% of the causes of death were due to NCDs in both men and women^(5)^. Among these, diabetes mellitus and endocrine disorders were responsible for 6.3% of the causes of death, being higher in females^(5)^. On the other hand, arterial hypertension promoted about 2.5% of the causes of death, also being higher in females. An important additional information presented by the World Health Organization (WHO) data platform is that, regardless of the age group, females were percentage-wise more affected by deaths due to diabetes mellitus, endocrine disorders, and arterial hypertension^(5)^. However, the data from the WHO health platform is limited up to the age group of 75 years, not presenting data on prevalence, risk factors, and not being specific for the population of very old elderly individuals (age 80 years and over).

Knowing that understanding the prevalence can be important information for better intervention in this elderly population, elderly individuals with NCDs, for example, have a higher risk of developing comorbidities associated with aging, such as osteoporosis, chronic kidney disease, peripheral vascular disease, metabolic dysfunction, frailty, sarcopenia, reduced gastrointestinal motility, postural hypotension, vascular dementia, coronary artery disease, vascular calcification, stroke, and arterial hypertension^(6-7)^.

In the United States, for instance, hypertension affects more than 75% of people aged 75 years or older^(8)^. However, it is known that prevalence varies according to sex and age, with women being more affected (74.4%) than men (65.6%) in the age group over 70 years, regardless of whether the country is developed or underdeveloped^(3)^. Moreover, according to the Surveillance of Risk and Protective Factors for Chronic Diseases by Telephone Survey (from portuguese VIGITEL), in 2019, in the 27 cities of Brazil^(9-10)^, the frequency of medical diagnosis of hypertension was 52% for men and 61% for women, respectively, aged 65 years or older.

Considering the previously cited information, unfortunately, hypertension presents a common metabolic pathway with diabetes mellitus, occurring simultaneously and sharing the same risk factors^(11-12)^. The coexistence of these two diseases in the same individual is not a coincidence, particularly in the presence of obesity and insulin resistance^(13)^.

It is concerning to consider the potential growth of diabetes mellitus associated with aging^(14)^. Worldwide, in 2019, healthcare expenditures related to this disease were higher in the age groups of 60 to 69 years (177 billion dollars), followed by the age groups of 50 to 59 years (173 billion dollars) and 70 to 79 years (171 billion dollars)^(4)^. Furthermore, healthcare expenditures in 2019 were higher for females than for males, and the estimate of expenses will increase in the years 2030 and 2045^(4)^.

Risk factors such as age and obesity, both associated with an increase in the quantity of senescent cells caused by chronic, systemic, and low-level inflammation, fibrosis, and organelle dysfunction (lipotoxicity, mitochondrial, and autophagy), are responsible for the development of diabetes and its complications^(6)^. For diabetes mellitus, according to VIGITEL^(9-10)^, in 2019, the frequency of medical diagnosis was 24% for men and 22% for women, respectively, aged 65 years or older in Brazil^(9-10)^.

Therefore, given that hypertension and diabetes mellitus are risk factors for congestive heart failure and stroke^(13,15)^ and represent about 2.5% to 6.3% of the causes of death in older adults aged over 75 years^(5)^, the identification of the prevalence and its risk factors in long-lived elderly people is crucial, especially since the percentage of deaths due to NCDs is higher in females. Risk factors for NCDs are generally classified into two groups: modifiable behavioral risk factors and metabolic risk factors^(16)^. The first group includes harmful use of alcohol, tobacco, physical inactivity, sedentary behavior^(17)^, and an unhealthy diet. Metabolic risk factors include increased blood pressure, overweight, obesity, hyperglycemia, and hyperlipidemia^(16)^.

Moreover, excess weight, which represents an important risk factor for diabetes mellitus, hypertension, and polypharmacy^(6,18-20)^, affects more than 50% of older adults aged 65 years or older in Brazil^(9-10)^. Considering that the prevalence of polypharmacy is higher in obese older adults compared to non-obese older adults and that the number of medications used is higher in older adults with a history of falls and low muscle quality^(20-22)^, the relevance of analyzing these issues is justified not only to ensure good living conditions for individuals but also because long-lived elderly people represent a particular challenge in gerontology, as this population, by 2050, will reach impressive numbers of 434 million, tripling the quantity compared to 2015^(1-2)^.

To date, long-lived elderly people are underrepresented in epidemiological studies, and in Brazil, there are few initiatives to understand the prevalence and risk factors associated with NCDs in this specific population.

## OBJECTIVE

To assess the prevalence of non-communicable chronic diseases, arterial hypertension, diabetes mellitus, and associated risk factors in long-lived elderly individuals from three Brazilian regions: Taguatinga (DF), Passo Fundo (RS), and Campinas (SP).

## METHODS

### Ethical aspects

Ethical aspects were observed according to Resolution No. 466 of December 12, 2012, of the National Research Council, which defines regulatory norms for research involving human beings. The study was conducted in accordance with national and international ethical guidelines and was approved by the Research Ethics Committee of each university that hosted the investigation: *Universidade de Passo Fundo - UPF* (Opinion No. 2.097.27/2017), *Universidade Católica de Brasília - UCB* (Opinion No. 1.290.368/2015), and *Universidade Estadual de Campinas* - UNICAMP (Opinion No. 3.061.534/2018), whose opinions are attached to this submission. This study was conducted in accordance with the STROBE (Strengthening the Reporting of Observational Studies in Epidemiology) manual for observational studies in epidemiology^(25)^. Informed consent was obtained from all individuals involved in the study through written means.

### Design, period, and location of the study

This is an observational, cross-sectional, analytical, and quantitative study conducted with long-lived elderly individuals aged 80 years or older. This research derives from a multicenter study called “Patterns of Physical, Cognitive, and Psychosocial Aging in Long-Lived Elderly Individuals Living in Different Contexts” (PROCAD), conducted from January 2016 to December 2018. The Gerontology Postgraduate Programs of the UNICAMP, São Paulo, *Universidade Católica de Brasília*, Distrito Federal, and the *Universidade de Passo Fundo*, Rio Grande do Sul, participated in this interinstitutional cooperation.

### Sample, inclusion and exclusion criteria

The sample was obtained from the electronic database of the PROCAD study, which included elderly individuals aged 80 years or older recruited from family households, long-term care institutions, and geriatric clinics in three Brazilian regions: Taguatinga (DF) with 196 elderly individuals, Passo Fundo (RS) with 272 elderly individuals, and Campinas (SP) with 232 elderly individuals. Men and women aged 80 years or older who did not have auditory and/or visual deficits and who were capable of comprehending and fully responding to the applied questionnaires and instruments were included in the study. To evaluate the comprehension and understanding of the questionnaires, the elderly individuals should have presented adequate levels of temporal orientation, spatial orientation, immediate memory, command, and reading ability assessed by the Mini-Mental State Examination (MMSE)^(26-27)^.

On the other hand, elderly individuals classified with cognitive deficits by the MMSE^(26-27)^, those who presented inability to maintain orthostatism with or without assistance, those with physical disabilities that prevented independent walking, such as lower limb amputations, self-reported diagnosis of stroke, depression (under treatment), vestibular or neurodegenerative diseases such as Alzheimer’s or other dementias, Parkinson’s disease, and any other disease that impaired the elderly’s mobility were excluded. A total of 19 elderly individuals were excluded in Campinas, 18 in Passo Fundo, and 14 in Taguatinga. After a subsequent analysis of the database containing the information of these participants, all those who presented incomplete records of the variables necessary for this research were excluded.

### Study protocol

Sociodemographic and clinical variables were evaluated through a face-to-face interview, using a questionnaire subdivided into blocks, composed of NCDs - heart disease, lung disease, systemic arterial hypertension (SAH), stroke, diabetes mellitus (DM), cancer, osteoporosis, osteoarthritis, and polypharmacy (considering the use of 5 or more medications)^(28-29)^. The questionnaires used were adapted by the researchers for collecting basic identification information. In addition, questions related to pre-existing disease diagnosis and number of medications evaluated by the geriatrician were collected, according to the information provided by the elderly individuals or their caregivers.

The anthropometric evaluation consisted of measuring body mass, height, body mass index (BMI), and waist circumference (WC). The elderly participants were weighed and measured using a digital electronic scale with a stadiometer and a capacity of 300 kg (Welmy® W300 brand). Height was measured after the oldest participant took a deep breath and stood completely erect. BMI was calculated as the ratio of body mass (kg) to height squared (m^2^) and classified according to the Ministry of Health recommendations for the elderly: underweight (BMI < 22 kg/m^2^), normal weight (BMI 22 kg/m^2^ - 27 kg/m^2^), and overweight (BMI > 27 kg/m^2^)^(30)^. WC was measured with an inelastic measuring tape, using as reference the midpoint between the iliac crest and the last rib. The cut-off points for cardiovascular risk in women were ≥ 88 cm, and for men were ≥ 102 cm^(31)^.

### Data analysis and statistics

Descriptive analysis included calculating frequency distributions and measures of central tendency and dispersion. Bivariate analysis was performed using the Chi-square test/Fisher’s exact test, with NCDs (heart disease, hypertension, stroke, and type 2 diabetes mellitus) as the dependent variable and socioeconomic factors (gender, age group, education, and income), polypharmacy use, and nutritional status (BMI and WC classification) as the explanatory variables. Predictive variables with p-values less than 20% were entered into the multivariate Poisson regression model with robust variance using the backward method, and the least significant variables (with the highest p-value) were removed one by one until all variables in the model had statistical significance (p < 0.05). The Hosmer & Lemeshow test was used to verify the fit of the final model.

The prevalence ratio (PR) with a 95% confidence interval (CI 95%) was used as the effect measure. It is noteworthy that since no explanatory variable had a p-value less than 20% in the bivariate analysis to analyze the factors associated with heart disease and stroke, no multivariate regression models were performed for these two diseases. Multivariate regression models were only performed for hypertension and type 2 diabetes mellitus. Data were analyzed using Stata version 11.0 software. For all analyses, a significance level of p ≤ 0.05 was adopted.

## RESULTS

### Sample characterization

A total of 700 elderly individuals were evaluated, with a mean age of 85.7 ± 4.8 years and 72.9% of them were female. It was observed that the majority of the elderly had up to 4 years of education (72.4%) (Table 1). The prevalence of overweight was 37.2% and 58.1% had risk of metabolic complications according to WC classification. The prevalence of hypertension, stroke, and type 2 diabetes mellitus was 13.3% and 25.9%, respectively (Table 1).

**Table 1:** Sample characterization

Variables	n	%
Sex		
Male	190	27.1
Female	510	72.9
Age group		
80-84	336	48.1
85-89	220	31.5
90 or more	143	20.5
Education		
Illiterate	95	22.5
Up to 4 years of study	211	49.9
From 5 to 8 years of study	55	13.0
More than 8 years of study	62	14.7
Family income in salary ranges		
< 1.0	58	14.3
1.1 to 3.0	174	42.9
>3.0		42.9
Polypharmacy (use of 5 or more medications)	383	59.5
BMI (classification)		
Low weight	131	21.4
Eutrophy	252	41.2
Overweight	228	37.3
Waist circumference (classification)		
Without risk	238	41.9
At risk of metabolic complications	330	58.1
Self-reported diseases		
Hypertension	454	68.2
Diabetes	170	25.9

Notes: Frequency; % - percentage.

### Prevalence of NCDs

Bivariate analysis (Table 2) revealed higher prevalence of hypertension among those who use polypharmacy (75.7% vs. 58.4%, p < 0.001), those who are overweight according to BMI classification (78.2% vs. 57.8% underweight and 66.8% normal weight), and those who have a risk of metabolic complications according to WC classification (73.8% vs. 61.2%, p = 0.002).

**Table 2:** Prevalence of heart disease, hypertension, stroke, and type 2 diabetes mellitus according to sociodemographic data, use of polypharmacy, and nutritional status of long-lived elderly individuals (N = 700)

Variáveis	Doenças do coração		Valor de *p* ^*^	AVE		Valor de *p* ^*^	Hipertensão arterial		Valor de *p* ^*^	Diabetes Mellitus tipo 2		Valor de *p* ^*^
	Sim (%)	Não (%)		Sim (%)	Não (%)		Sim (%)	Não (%)		Sim (%)	Não (%)	
Sex			0.235			0.464			0.060			0.119
Male	25.6	74.4		15.0	85.0		62.4	37.6		30.4	69.6	
Female	21.2	78.8		12.7	87.3		70.2	29.8		24.3	75.7	
Age group			0.826			0.220			0.060			<0.001
80-84	23.4	76.6		11.1	88.9		72.6	27.4		33.9	66.1	
85-89	21.2	78.8		16.4	83.6		64.8	35.2		20.3	79.7	
90 or more	21.8	78.2		13.6	86.4		63.0	37.0		16.5	83.5	
Education			0.521			0.669			0.326			0.735
Illiterate	22.9	77.1		12.3	87.7		80.7	19.3		31.8	68.2	
Up to 4 years of study	22.6	77.4		7.8	92.2		73.0	27.0		28.6	71.4	
From 5 to 8 years of study	15.9	84.1		11.1	88.9		79.6	20.4		26.5	73.5	
More than 8 years of study	28.6	71.4		9.1	90.9		69.5	30.5		35.1	64.9	
Family income in salary ranges			0.068			0.946			0.394			0.423
< 1.0	32.0	68.0		10.6	89.4		83.0	17.0		22.0	78.0	
1.1 to 3.0	18.1	81.9		9.1	90.9		75.0	25.0		30.1	69.9	
>3.0	26.6	73.4		9.8	90.2		73.9	26.1		31.6	68.4	
Polypharmacy (use of 5 or more medications)			<0.001			<0.001			<0.001			<0.001
Yes	27.5	72.5		18.2	81.8		75.7	24.3		33.0	67.0	
No	14.1	85.9		6.5	93.5		58.4	41.6		15.8	84.2	
BMI (classification)			0.975			0.802			<0.001			0.722
Low weight	22.0	78.0		13.7	86.3		57.8	42.2		23.4	76.6	
Eutrophy	21.0	79.0		12.0	88.0		66.8	33.2		25.4	74.6	
Overweight	21.5	78.5		11.3	88.7		78.2	21.8		27.3	72.7	
Waist circumference (classification)			0.612			0.907			0.002			0.005
Without risk	22.1	77.9		11.4	88.6		61.2	38.8		19.9	80.1	
At risk of metabolic complications	20.3	79.7		11.0	89.0		73.8	26.2		30.7	69.3	

* Chi-square test/Fisher’s exact test; % - percentage; Stroke - Cerebrovascular accident.

In bivariate analysis (Table 2), higher prevalence of diabetes were observed among older adults in the younger age group (33.9% among those aged 80 to 84 years, 20.3% among those aged 85 to 89 years, and 16.5% among those aged 90 years or older, p < 0.001), those who use polypharmacy (33.0% vs. 15.8%, p < 0.001), and those who have a risk of metabolic complications according to WC classification (30.7% vs. 19.9%, p = 0.005).

### Predictors of NCDs

According to the multivariate model (Table 3), the factors that presented independent association with arterial hypertension in elderly individuals were age group, polypharmacy, and BMI. The prevalence of hypertension was 22% (PR: 0.88; 95% CI: 0.78-0.99) and 29% (PR: 0.83; 95% CI: 0.71-0.97) lower among individuals aged 85 to 89 years and 90 years or older when compared to those aged 80 to 84 years; it was 38% higher among elderly individuals who use 5 or more medications (PR: 1.38; 95% CI: 1.23-1.55); and 17% higher among elderly individuals with overweight (PR: 1.17; 95% CI: 1.05-1.30) when compared to those with normal weight.

**Table 3:** Multiple model of association between selected variables and self-reported arterial hypertension in long-lived elderly individuals

Variables	Arterial hypertension		*p* value^*^
	PR	CI 95%	
Age group			
80-84	(ref.)	-	-
85-89	0.88	0.78-0.99	0.045
90 or more	0.83	0.71-0.97	0.020
Polypharmacy (use of 5 or more medications)			
Yes	1.38	1.23-1.55	<0.001
No	(ref.)	-	-
BMI (classification)			
Low weight	0.86	(1.05-1.30)	0.084
Eutrophy	(ref.)	-	-
Overweight	1.17	1.05-1.30	0.003

* Multiple regression model; CI - Confidence interval; PR - Prevalence ratio

According to the multivariate model (Table 4), the factors that presented independent association with type 2 diabetes mellitus in elderly individuals were sex, age group, and polypharmacy. The prevalence of diabetes was 24% (PR: 0.76; 95% CI: 0.59-0.98) lower among women when compared to men; it was 44% (PR: 0.56; 95% CI: 0.42-0.74) and 57% (PR: 0.43; 95% CI: 0.29-0.65) lower among individuals aged 85 to 89 years and 90 years or older when compared to those aged 80 to 84 years; and 2.15 times higher among elderly individuals who use 5 or more medications (PR: 2.15; 95% CI: 1.63-2.85).

**Table 4:** Multiple model of association between selected variables and type 2 diabetes mellitus in elderly individuals

Variables	Diabetes Mellitus Type 2		*p* value^*^
	PR	CI 95%	
Sex			
Male	(ref.)	-	-
Female	0.76	0.59-0.98	0.041
Range age			
80-84	(ref.)	-	-
85-89	0.56	0.42-0.74	<0.001
90 or more	0.43	0.29-0.65	<0.001
Polypharmacy (use of 5 or more medications)			
Yes	2.15	1.63-2.85	<0.001
No	(ref.)	-	-

* Multiple regression model; CI - Confidence interval; PR - Prevalence ratio.

## DISCUSSION

The results of the present study demonstrated higher prevalence of arterial hypertension among those who use polypharmacy, among the elderly aged 80-84 years, and higher prevalence among those who presented overweight and risk of metabolic complications according to the WC classification. In addition, the risk factors that presented independent association with arterial hypertension were age group, polypharmacy, and BMI.

For type 2 diabetes mellitus, higher prevalence was observed among elderly people aged 80-84 years, those who used polypharmacy, and those who presented risk of metabolic complications according to the WC classification. The prevalence of type 2 diabetes mellitus was 24% lower among women when compared to men and 2.15 times higher among those who use 5 or more medications. Finally, the risk factors that presented independent association with type 2 diabetes mellitus were gender, age group, and polypharmacy.

The higher prevalence of arterial hypertension and type 2 diabetes mellitus demonstrated in the present study corroborates with the results of the literature^(3-4,17)^, being hypertension and diabetes commonly presented as the main comorbidities and positively correlated with advancing age^(3-4,32-33)^. Despite this, for the long-lived elderly people of the present study, a higher prevalence of the diseases was not verified in the female sex as pointed out by previous studies^(3-4)^.

Regarding the prevalence of diabetes mellitus in long-lived elderly people, a previous study^(34)^ found results similar to the present study. Recently, the authors observed that the prevalence for type 2 diabetes mellitus increased with age and then decreased in participants over 80 years old when compared to younger elderly people (60 years; 25.5% versus 56.9%)^(34)^. In addition, the higher prevalence of the disease in males compared to females found in the present study does not corroborate with the latest global statistical data^(4)^. However, according to VIGITEL, in the year 2019^(9-10)^, men aged 65 years or older presented higher prevalence when compared to women, but there are no comparative data for long-lived elderly people. Therefore, it is important to understand that data on prevalence in long-lived elderly people are still scarce and inconclusive.

One of the possible hypotheses for the lower prevalence of diabetes mellitus in women is that they are more inclined to pay attention to symptoms and seek regular medical care compared to men, as well as being more involved in preventive activities and living a healthier lifestyle^(34)^. In other words, they have a different attitude towards diseases and the concept of health^(35-36)^. However, a previous study showed no association between sex and the prevalence of diabetes and hypertension in Brazilian elderly individuals^(37)^.

However, a risk factor that accompanies these NCDs is polypharmacy^(38)^. Although the definition of polypharmacy varies in the literature in relation to the number of medications used^(23,38)^, an increase in medication use is demonstrated according to age group. Being 36% for participants aged 75 to 84 and 46% for participants aged 85 or more^(28)^. However, this data is worrisome, especially for the long-lived elderly population in this study, as the age group of 85 years or more, presence of six or more chronic health conditions, low body weight and BMI, and frailty should be taken into account as they represent important risk factors for adverse drug events such as falls, hospitalization, sedation, depression, and mortality^(23,38)^.

Furthermore, long-lived elderly individuals who have already been affected by a disease often use medications for treatments associated with dysfunctions in different bodily systems, such as the nervous and gastrointestinal systems, for example (e.g. hypnotics, sedatives, anxiolytics, antipsychotics, and anti-ulcer agents)^(23,38-39)^. This makes it understandable the association between a higher number of diagnoses of diseases or multiple diseases (not evaluated in this study) and polypharmacy^(40)^. Moreover, supporting data from previous studies, obesity, evaluated in this study and identified as a risk factor, using obesity indices easily applied in clinical practice (e.g. BMI and WC), is associated with increased medication use and may result in chronic diseases^(6,20,34,41)^, especially with advancing age^(20,33,37,42-43)^.

### Study limitations

Although the present study presents interesting and innovative results for the long-lived elderly people who participated in it, important limitations should be cited. Among them, variables such as demographic region, skin color/race, education, smoking, and physical inactivity should also be considered in subsequent analyses of the prevalence and risk of NCDs in long-lived elderly people^(17,37)^, as they are related to diabetes and hypertension. In addition, the inclusion of elderly people from the community and long-term care institutions in the present study may represent an important bias in the prevalence and risk factors for NCDs in the results presented, as they may have distinct life and health profiles.

For example, in a previous study of institutionalized elderly people in the municipality of Passo Fundo, Rio Grande do Sul, the majority were female (63.2%), 48% were long-lived elderly people, 96.4% used medications, and 74% were in the institution because they needed care^(44)^. Furthermore, our findings should be validated in longitudinal studies with large samples. Finally, the cross-sectional design of the study precludes the ability to infer any causal relationship.

### Contributions to the nursing, health, and public policy areas

Our findings may contribute to directing the interprofessional evaluation of the elderly person, aiming for greater efficacy in the elaboration of care plans that aim at better evaluation of the critical factors that interfere with NCDs. Understanding the number of medications used, body weight gain, and specific gender characteristics enables the development of more targeted practices, treatments, and policies for professionals who work with different age groups of the elderly.

Considering the results obtained in the present epidemiological study, nurses should pay attention to the continued care of elderly individuals with less health, either due to the circumstances of their morbidity or due to the natural process of frailty in the face of greater longevity, providing means to help maintain their quality of life.

## CONCLUSION

In the three Brazilian regions evaluated in this study (Taguatinga, Passo Fundo, and Campinas), higher prevalence of hypertension were found in elderly individuals aged 80-84 years. Additionally, the prevalence of diabetes was lower in women compared to men but higher among those who use more medications and in the age group of 80-84 years. Among the important risk factors for hypertension and diabetes mellitus, overweight and polypharmacy represented two important variables in the sample of long-lived elderly individuals in the present study.

## Data Availability

https://doi.org/10.48331/scielodata.LUGU4D
